# Supplementary figures and images for: Integration of biological data via NMF for identification of human disease-associated gene modules through multi-label classification
Source: PLoS One. 2024 Dec 12;19(12):e0305503. doi: 10.1371/journal.pone.0305503 (PMC11637261; doi:10.1371/journal.pone.0305503)

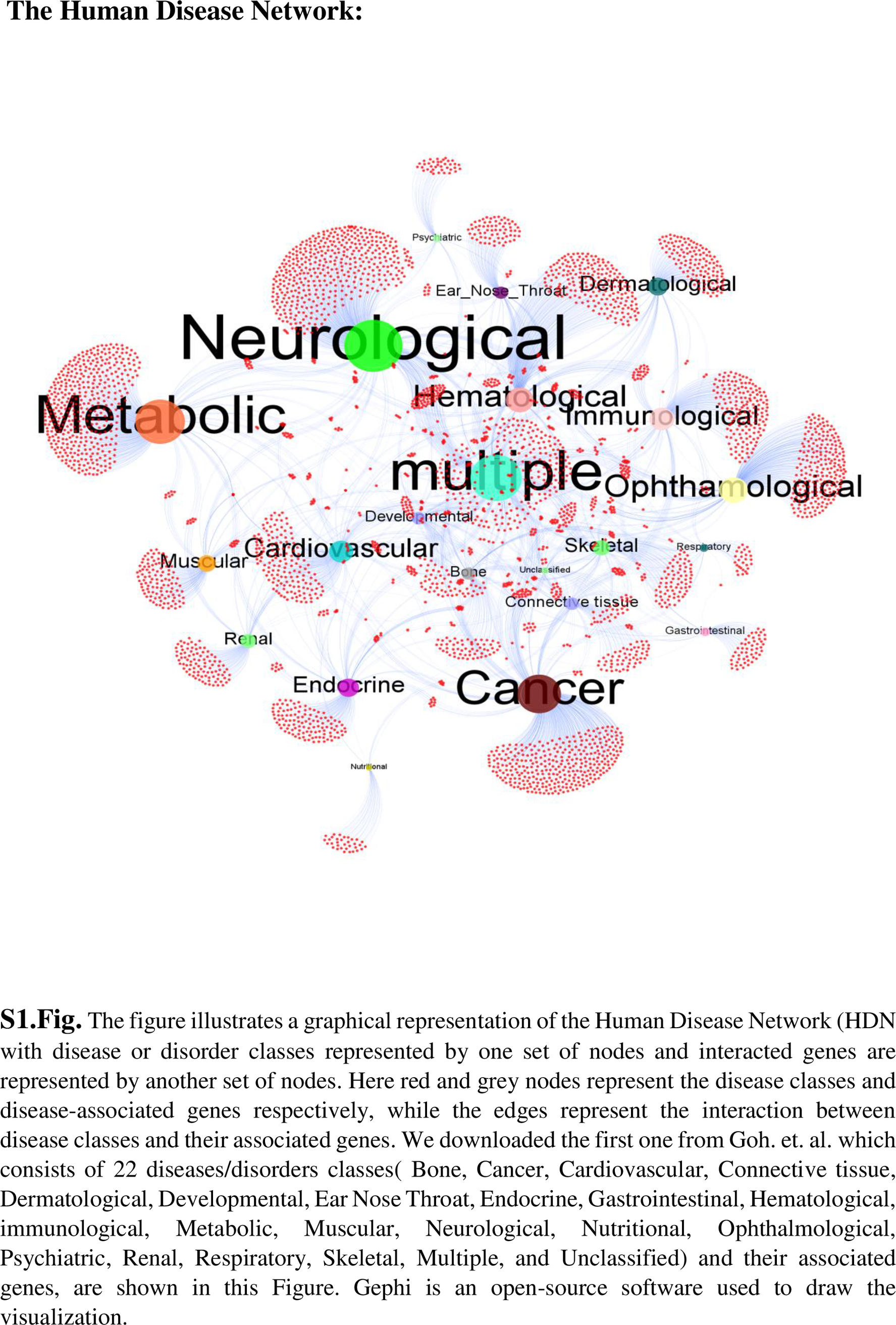

Supplement: S1 Fig — The figure illustrates a graphical representation of the Human Disease Network (HDN with disease or disorder classes represented by one set of nodes and interacted genes represented by another set of nodes. (TIF) [file pone.0305503.s001.tif]

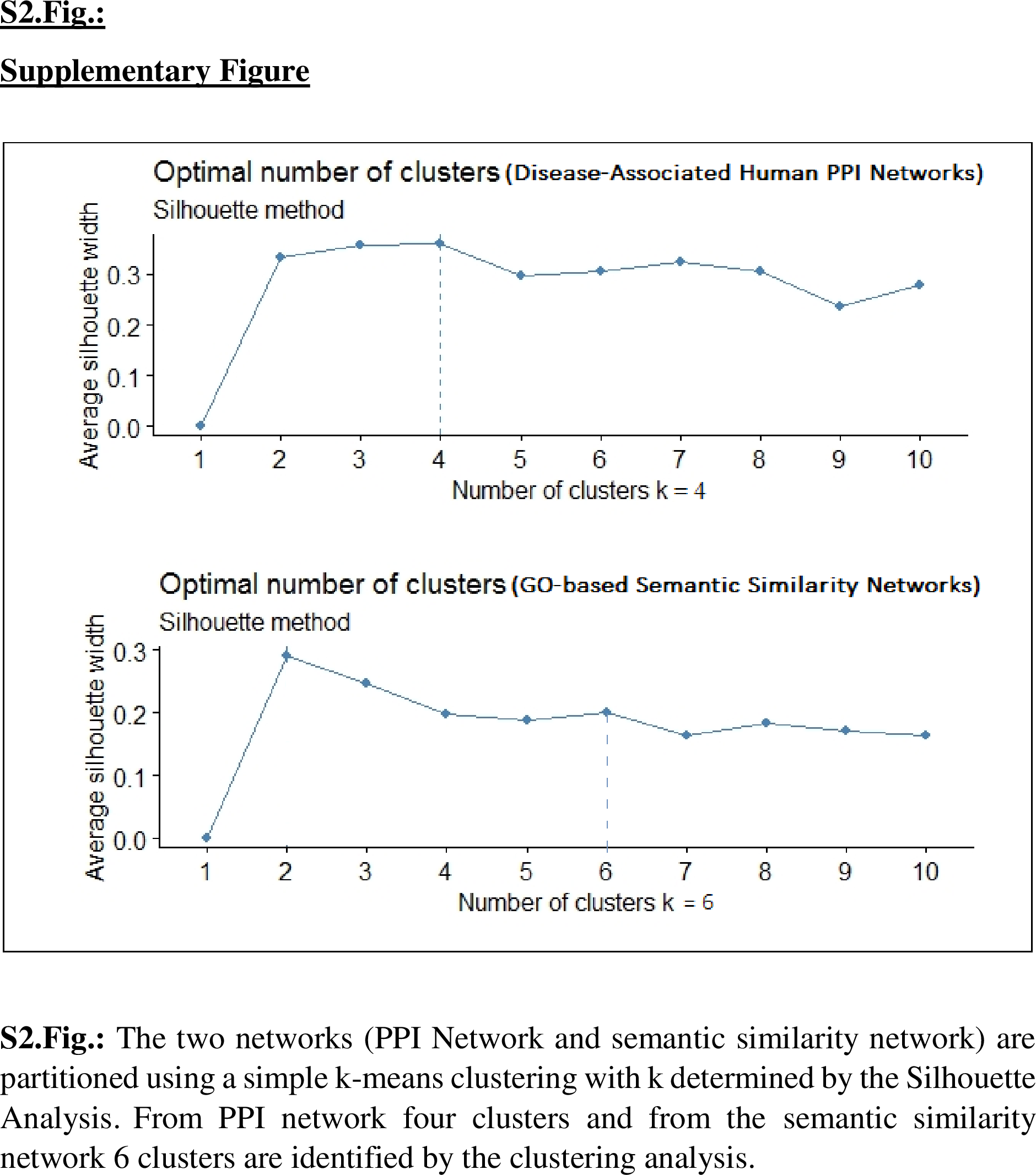

Supplement: S2 Fig — The two networks (PPI Network and semantic similarity network) are partitioned using a simple k-means clustering with k determined by the Silhouette Analysis. (TIF) [file pone.0305503.s002.tif]

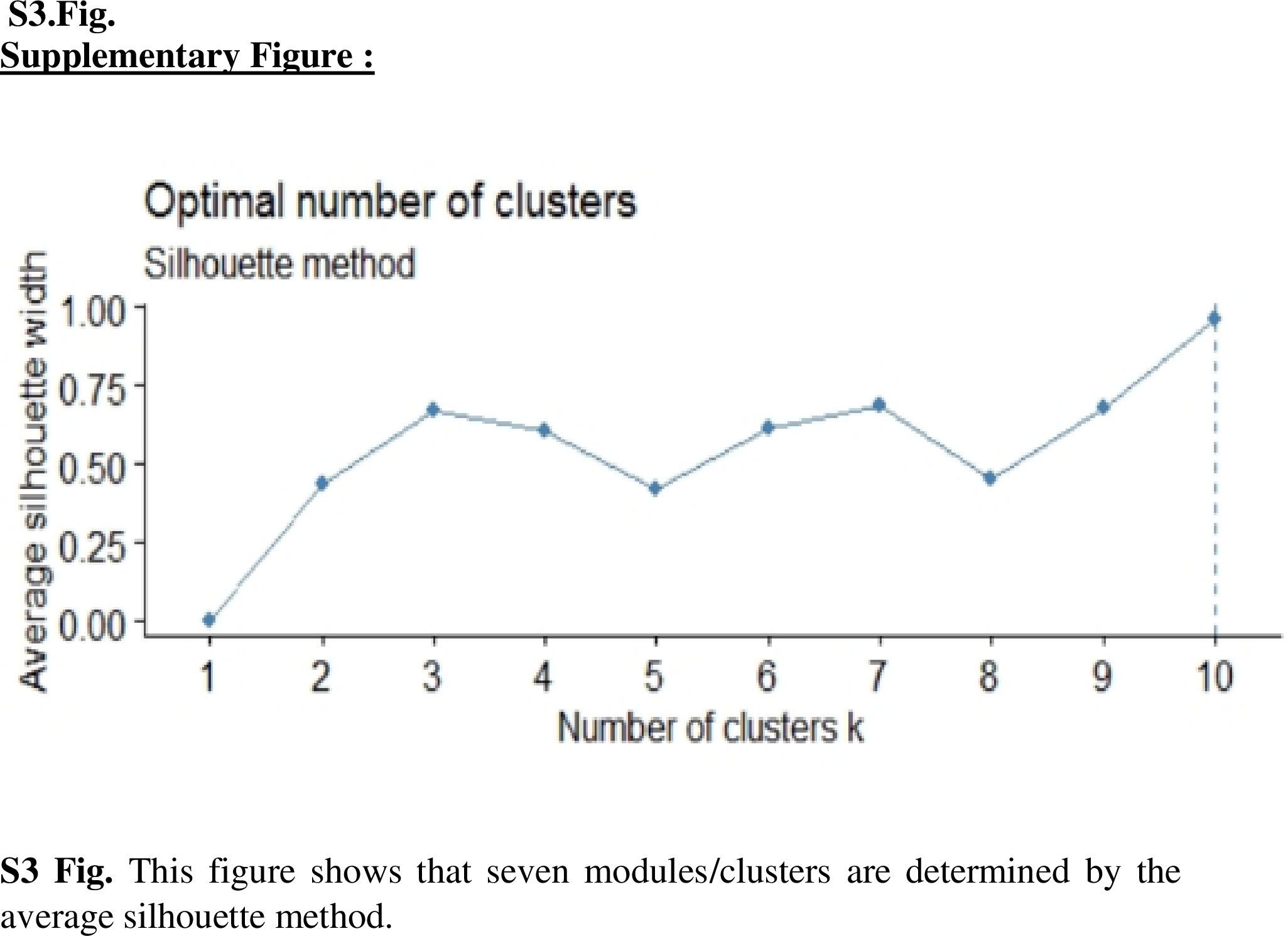

Supplement: S3 Fig — (TIF) [file pone.0305503.s003.tif]

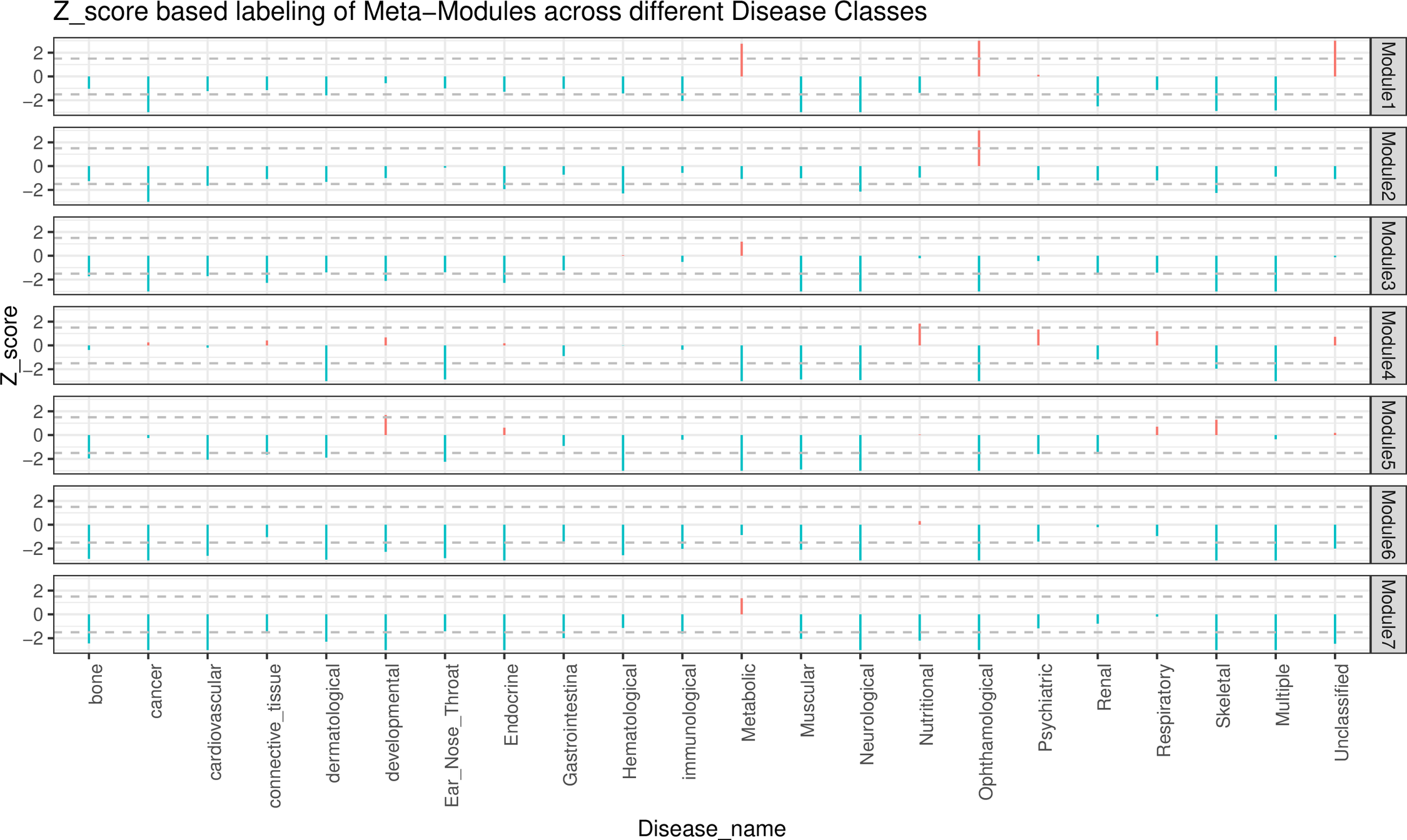

Supplement: S4 Fig — (TIF) [file pone.0305503.s004.tif]

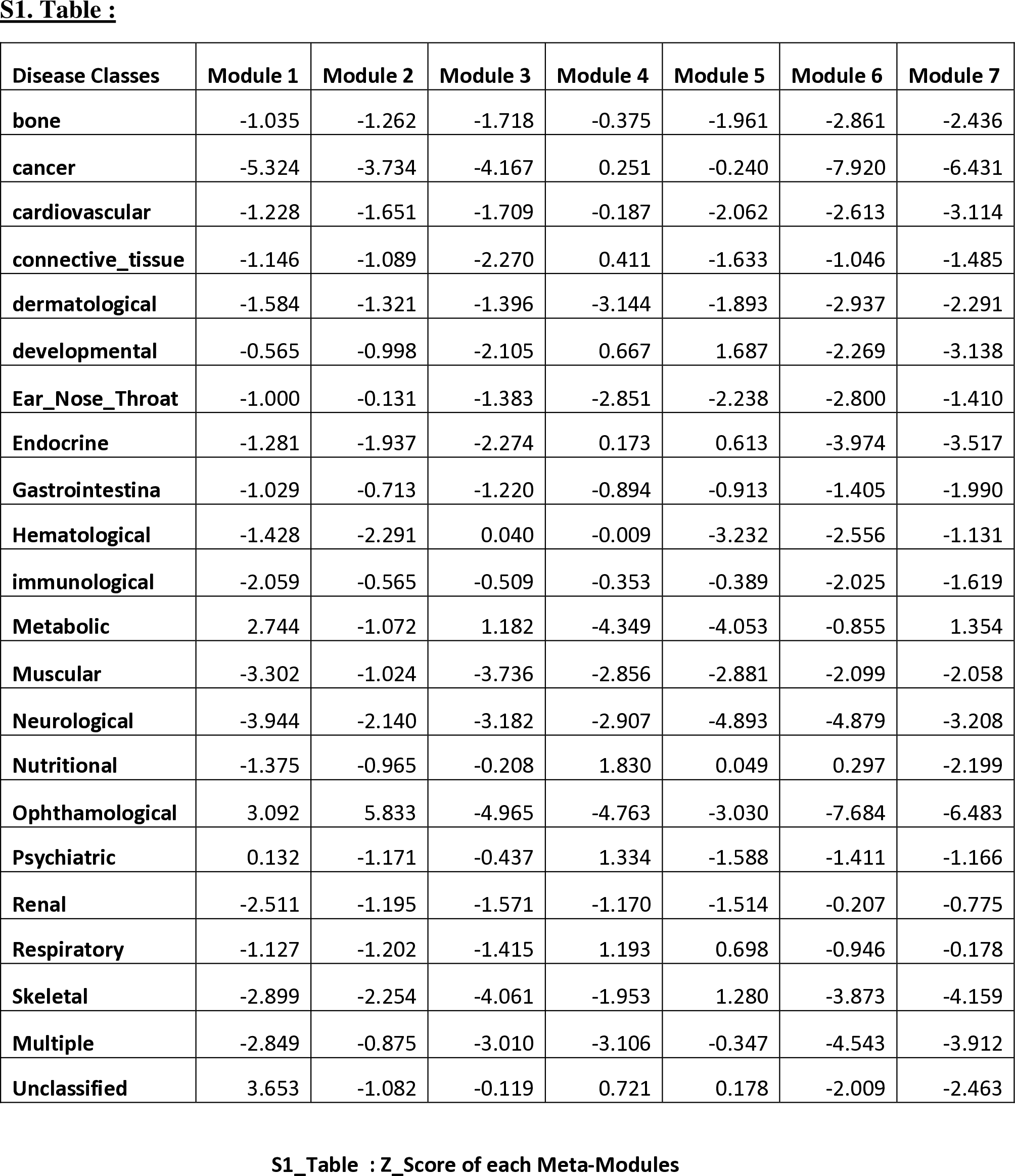

Supplement: S1 Table — (TIF) [file pone.0305503.s005.tif]
